# The Role of Thoracic Endovascular Aortic Repair (TEVAR) of Thoracic Aortic Diseases in Patients with Connective Tissue Disorders - A Literature Review

**DOI:** 10.21470/1678-9741-2019-0367

**Published:** 2020

**Authors:** Amer Harky, Syed Mohammad Asim Hussain, Beverly MacCarthy-Ofosu, Mohammad Usman Ahmad

**Affiliations:** 1 Department of Cardiothoracic Surgery, Liverpool Heart and Chest, Liverpool, UK.; 2 School of Medicine, University of Liverpool, Liverpool, UK.; 3 Department of Surgery, Hull University Teaching Hospitals, Hull, UK.

**Keywords:** Aorta, Thoracic, Dilatation, Aneurysm, Dissecting, Aortic Diseases, Endovascular Procedures

## Abstract

**Objective:**

To review the currently available literature to define the role of thoracic endovascular aortic repair (TEVAR) in patients with connective tissue disorders (CTD).

**Methods:**

A comprehensive electronic database search was performed in PubMed, SCOPUS, Embase, Google scholar, and OVID to identify all the articles that reported on outcomes of utilizing TEVAR in patients with CTD during elective and emergency settings. The search was not limited to time or language of the published study.

**Results:**

All the relevant studies have been summarized in its correspondence section. The outcomes were analyzed in narrative format. The role of TEVAR has been elaborated as per each study. Currently, there is limited large cohort size studies outlining the use of TEVAR in patients with CTD. The use of endovascular repair in patients with CTD is limited due to progressive aortic dilatations and high possibility of further reinterventions at later stage of life.

**Conclusion:**

Open repair remains the gold standard method of intervention in young patients with progressive CTD, especially in the setting of acute type A aortic dissection. However, TEVAR can be sought as a reliable alternative in emergency setting of diseases involving the descending thoracic aorta; yet the long-term data needs to be published to support such practice.

**Table:** 

Abbreviations, acronyms & symbols	
**CT** **CTD** **EACTS** **EDS** **FBN1** **INSTEAD** **LDS** **MFS** **MR** **TAA** **TEVAR** **TGFB**	**= Computed tomography** **= Connective tissue disorders** **= European Association for Cardio-thoracic surgery** **= Ehlers-Danlos syndrome** **= Fibrillin-1** **= INvestigation of STEnt Grafts in Aortic Dissection** **= Loeys-Dietz syndrome** **= Marfan syndrome** **= Magnetic resonance** **= Thoracic aortic aneurysm** **= Thoracic endovascular aortic repair** **= Transforming growth factor-β**

## INTRODUCTION

Connective tissue can be found throughout the body and is composed of proteins such as collagen and elastin. These proteins function to provide extracellular binding and support in various tissues and organs^[1]^. Inherited genetic abnormalities may lead to connective tissue disorders (CTD), which cause tissue degeneration and loss of structural integrity^[2]^.

The cardiovascular system is one of the most important organ systems that is adversely affected in patients with CTD. The aorta, which is exposed to high pressure from constant blood flow, becomes weakened; therefore, it is at increased risk of developing aneurysm, rupture, and dissection^[3]^. The most common CTDs which are associated with aortic disease are Marfan syndrome (MFS), vascular Ehlers-Danlos syndrome (EDS), and Loeys-Dietz syndrome (LDS)^[4]^. Aortic disease is the most prevalent cause of high morbidity and mortality in this patient population, and surgical repair is one of the main treatment options available^[5]^. Currently, open surgical repair is the recommended approach. This can be challenging in most of these patients, as the majority of such cohorts with CTD may require multiple aortic surgeries during their lifetime. Endovascular aortic repair has proven to have lower success rate in CTD patients and as such it is not considered standard practice^[5]^. This paper aims to review the currently available literature to define the role of endovascular thoracic aortic repair in patients with CTD. A comprehensive literature search was conducted in major electronic databases (PubMed, SCOPUS, Embase, and Google Scholar) to identify articles that discuss the outcome of utilizing thoracic endovascular aortic repair (TEVAR) in patients with CTD. No limits were placed on language, cohort size, or year of publication. All relevant articles in the acute and elective setting are tabulated as per each relevant section.

## CONNECTIVE TISSUE DISORDERS

The three key diseases that are affecting the aorta are MFS, vascular EDS, and LDS.

**Fig. 1:**
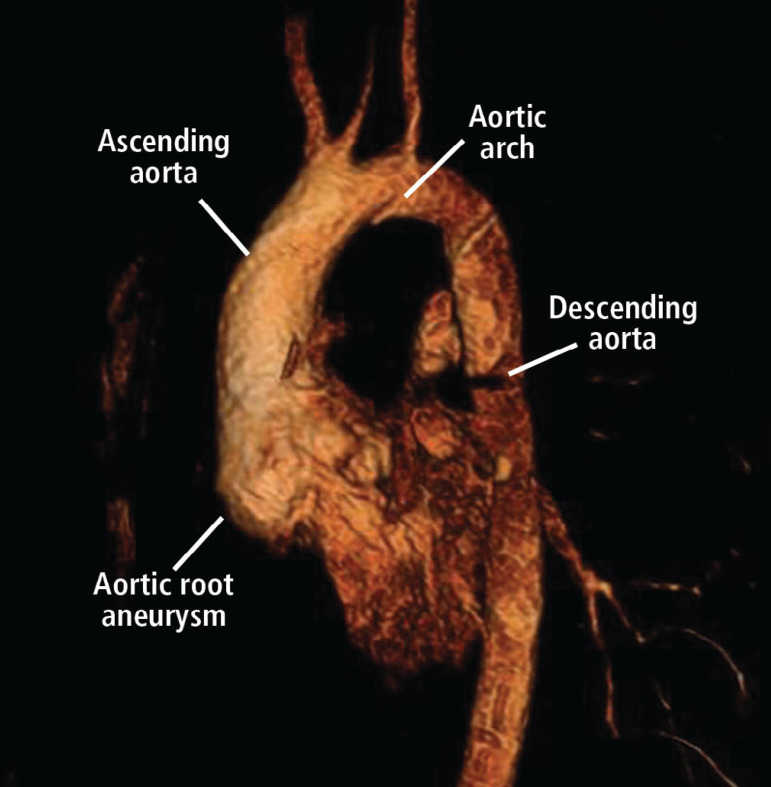
A three-dimensional computed tomography of aorta showing aortic root aneurysm in a patient with Marfan syndrome^[13]^.

**Fig. 2:**
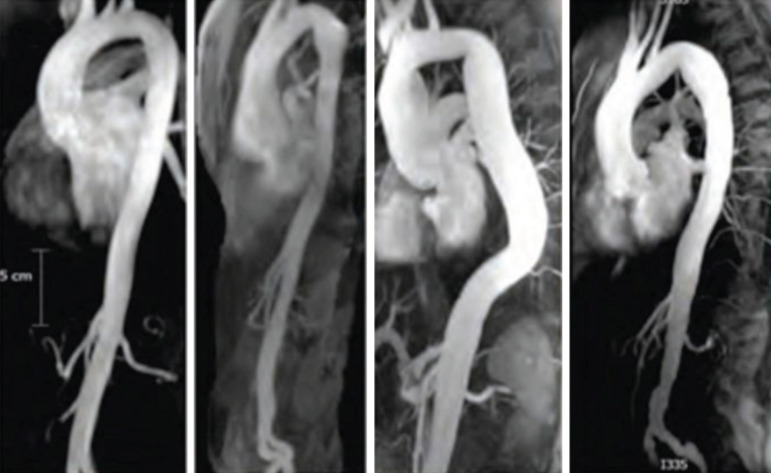
Aortic imaging of a patient with Marfan syndrome showing dilatation of the thoracic aorta^[14]^.

**Fig. 3:**
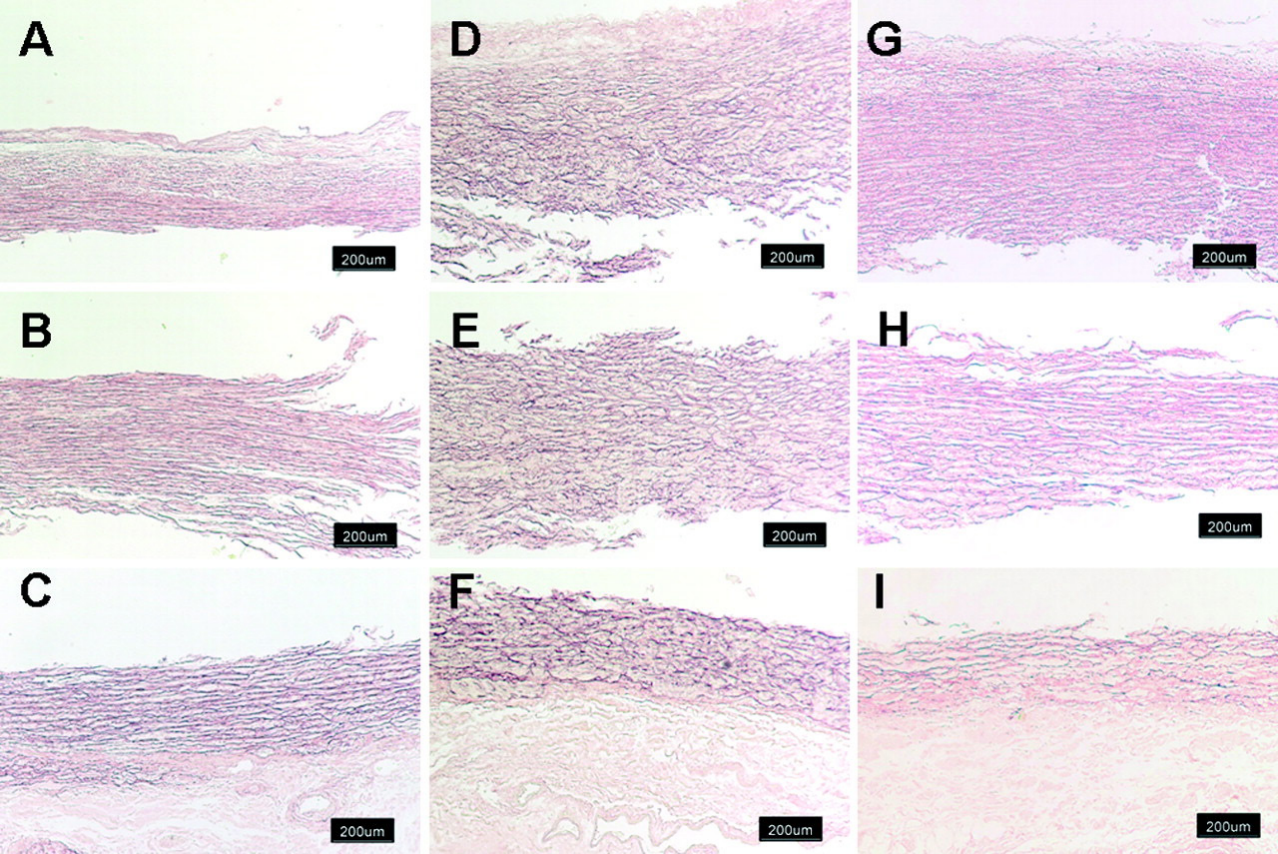
Histology and separation of aortic tissue for proteomic analysis. Van Gieson elastic stain of aorta from representative control (A through C) and aneurysmal aorta from Marfan syndrome (MFS) patients (D through F) and bicuspid aortic valve patients (G through I) are shown. Aorta from MFS patients presents fragmentation of elastic fibers and thickened aortic wall (magnification ×4). Intima with media (A, D, and G), separated media (B, E, and H), and media with adventitia (C, F, and I) are shown^[15]^.

**Fig. 4:**
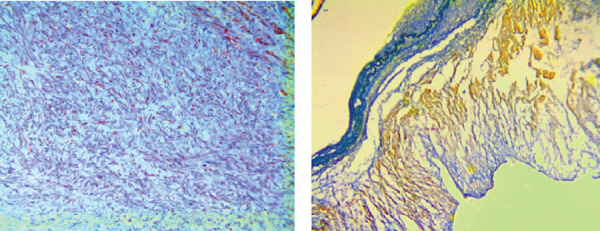
Marfan aortic tissue showing cystic medial necrosis with (A) smooth muscle fragmentation and more collagen deposition, Masson ´200; and (B) proliferation and disruption of the intima (blue), and smooth muscle cell fragmentation (yellow) and collagen deposition (red) in the media. VG-Victoria blue bichrome staining ´100^[16]^.

MFS affects approximately 2-3 in 10,000 people^[6]^ and is diagnosed according to a set of clinical findings known as the revised Ghent criteria^[7]^. It is caused by an autosomal dominant mutation in the fibrillin-1 (FBN1) gene. The FBN1 gene is located on chromosome 15q21.1 and is responsible for the production of proteins that form the extracellular matrix which mediate the attachment of smooth muscles to collagen and elastin fibers^[3,6]^. This increases the strength and structural integrity of the aortic walls. Transforming growth factor-β (TGFB) becomes overly active in the presence of the faulty FBN1 gene and causes inflammation and fibrosis of the aorta. This leads to aortic wall weakness, resulting in dilatation and aneurysm formation, which accounts for approximately 80% of morbidity in MFS patients^[6]^. The aortic annulus, root, and ascending aorta are the sites most frequently affected by progressive weakening^[8]^. Dilatation of the sinus of Valsalva starts during the intrauterine period^[9]^. As the patient grows, the increase in the aorta’s size makes it more likely to develop a rupture or dissection^[10]^. It is advised that adults with an aortic root diameter > 45 mm be considered for surgery. In children, there is no set diameter which calls for surgery. However, growth of more than 1 cm per year, issues of valvular insufficiency, and Z-score > 2-3 in aortic root diameter are used as a guide^[11]^. The average life expectancy of patients with MFS is about 60 years^[12]^. Figures 1 and 2 are scans showing aneurysms and descending thoracic dissection, while Figures 3 and 4 are histologies of aortic tissues in Marfan patients^[13-16]^.

On the contrary, EDS refers to a range of genetic disorders which affect collagen production; therefore, it results in joint and skin defects, as well as tissue fragility^[3]^. There are seven main classifications of EDS, with vascular EDS type IV^[11]^ being the most commonly implicated for cardiovascular complications. In vascular EDS, there is a mutation in the COL3A1 gene which encodes for type III procollagen^[17,18]^. Type III procollagen makes up the highest proportion of collagen in the aortic wall; therefore, its defect results in severe aortic wall fragility and increased risk of rupture^[18,19]^. Vascular EDS usually affects the descending or abdominal aorta^[20]^, and the risk of rupture, unlike MFS, can occur with any diameter^[20]^. The high incidence of vessel rupture reduces the life expectancy of vascular EDS patients to about 48 years, as opposed to 60 years in MFS patients^[20]^.

LDS is caused by a heterozygous mutation in the TGFBR1 and TGFBR2 genes, which encode TGFB receptors I and II, respectively. The mutation triggers an increase in collagen production, while reducing the production of elastin and disrupting the assembly of elastin fibers. LDS is categorized into two main groups, namely, type 1 and type 2. Both conditions can cause vascular diseases in the form of aortic aneurysm, arterial tortuosity, and aortic dissection^[21,22]^. Unlike MFS, in LDS, the aortic root is prone to rupture and dissection during childhood and at even smaller diameters. Aneurysms occur less frequently in LDS, at a rate of 9%^[23]^. However, the aorta is prone to have increased growth rate and can sometimes be as twice as its normal size in the thoracic region^[24]^. Close monitoring as well as early surgical intervention are advised to prevent catastrophic outcomes. However, the mean life expectancy for LDS patients remains low, at about 37 years, although sporadic cases have been reported to live as late as 70 years of age^[22,25]^.

### Management of Aneurysmal Diseases

A true aortic aneurysm is defined as a localized dilatation involving all three layers of the aorta. The diameter should be at least 1.5 times that of the normal diameter to be considered as true aneurysm. Thoracic aortic aneurysm (TAA) may occur in the aortic root/ascending aorta (60%), aortic arch (40%), and the descending aorta including the thoracoabdominal aorta (10%)^[26]^. The normal aortic diameter varies with the age, gender, and size of the general population. A diameter > 4.5 cm is considered aneurysmal in the United States of America, whereas a diameter > 4 cm is defined as aneurysmal in South Korea^[27]^.

TAAs are associated with CTD and can lead to aortic incompetence, rupture, and acute dissection. This is the leading cause of premature death in patients with MFS. The pathological hallmark of aneurysmal disease in CTD is cystic medial necrosis, which results from degenerative disruption of collagen, elastin, or smooth muscle, leading to weakening of the arterial wall^[28]^.

Early diagnosis and treatment before the development of life-threatening complications is the key to managing TAAs. Useful imaging modalities include chest X-rays, transthoracic and transoesophageal echocardiography, multidetector computed tomography (CT) of aorta, and magnetic resonance (MR) angiography. Clinically, contrast-enhanced CT of aorta is the standard imaging technique to diagnose aortic pathology with a sensitivity up to 100% and specificity up to 99%^[29]^.

A greater understanding of genetics and inheritance patterns has allowed screening for aortic disease. Consensus guidelines were developed in 2010 by the American College of Cardiology and the American Heart Association; the key recommendations include^[30]^:

First-degree relatives of patients with aortic dissection or TAAs should be screened with aortic imaging to identify asymptomatic disease.

MFS patients should have an immediate echocardiogram to assess the aorta and followed up in six months to check for enlargement.

Patients with LDS should have complete aortic imaging immediately and six months later to check for enlargement and annual MR imaging from cerebrovascular circulation to the pelvis.

Once a diagnosis of TAA is established in a patient with underlying connective tissue disease, the primary treatment option is open surgical repair. Another important aspect in the management of patients developing aortic pathology is hypertensive control. Hypertension will accelerate the risk and rate that pathology develops, therefore anti-hypertensives are important in the management of aortic aneurysms^[31]^. Endovascular repair is not recommended in this cohort as stated in 2012 position statement of the European Association for Cardio-thoracic surgery (EACTS), the European Association of Cardiology, and the European Association of Percutaneous Coronary Intervention. They state that TEVAR is not recommended in patients with connective tissue disease except as a bail-out procedure or bridge to definitive open surgery or as a procedure following previous aortic surgery where both landing zones lie within previously sited synthetic grafts^[31]^.

In deciding to treat TAA, the risk of the intervention must be balanced against the risk of rupture or dissection. Patients with aneurysms of 6 cm have a risk of yearly rupture or dissection of 6.9% and mortality risk of 11.8%. The risks increase significantly for aneurysms > 6 cm^[32]^. Current guidelines ponder the following factors^[33]^ in considering intervention:

Aortic dilatation > 5 cm in patients with MFS, which is a lower threshold when compared to the general population (> 5.5 cm) (level IC). In patients with family history of acute dissection, severe aortic or mitral regurgitation, and rate of increase of 3 mm/year, the threshold for surgery is lower, still with diameter > 4.5 cm (level IIC).

Women with MFS contemplating pregnancy may undergo aortic root and ascending aorta replacement at a diameter of 4 cm as per American and Asian guidelines (level IIC), while European guidelines recommend a diameter > 4.5 cm.

In patients with LDS, an aortic diameter > 4 cm for the aortic root, > 5 cm for the descending thoracic aorta, and rate of increase > 0.5 cm/year regardless of location[(34)].

### Emergencies in Patients with Connective Tissue Disorders

These encompass aortic dissection, intramural haematoma, and penetrating atherosclerotic ulcer with or without aortic rupture. All of these pathologies result in disruption of the tunica media. Aortic dissection is the result of an intimal tear which allows blood to flow and splits the inner two-thirds and outer one-third of the tunica media apart creating a false lumen for blood flow.

The key clinical feature is chest pain, which has sudden onset, is severe, and may be described as tearing, sharp, or stabbing. The pain’s location is indicative of the site of intimal disruption and may change as the dissection extends along the aorta and other vessels. Pain can be associated with an aortic regurgitation murmur, wide pulse pressures, or tamponade. Occasionally, the presentation can be similar to the acute coronary syndrome, with electrocardiogram changes and elevation of cardiac enzymes. Pain in the back suggests involvement of the descending aorta. Associated symptoms include vomiting, sweating, and lightheadedness, and in severe cases or delayed presentation, complications may occur in the form of stroke, spinal cord injury, or mesenteric ischaemia.

The management depends on the extent of aortic involvement as per the Stanford classification. Type A dissections are managed surgically through open repair, whereas type B dissections are initially managed medically but may require surgery if unstable or progressive.

The principles of surgical management for type A dissections include excision of the intimal tear, preventing entry of blood flow into the false lumen, and replacement of the diseased aorta by anastomosing a synthetic interposition graft. The coronary arteries may be reimplanted, and the aortic valve may be replaced or repositioned in the presence of aortic regurgitation.

### Interventions in Type A Dissection

There are different approaches for acute pathologies for each segment of the aorta; this is purely depending on site and extent of the disease. These include, but are not limited to, conservative repair of the root, replacement of the root and valve with a composite graft conduit or valve sparing root replacement, or in more extensive cases, it involves replacement of the aortic arch either in partial, as in hemiarch replacement, or in full, as in total arch replacement.

Aortic root repair can be done in the majority of type A dissections and it has benefits such as preservation of native tissue and avoidance of manipulation of coronary arteries, thereby requiring shorter cardiopulmonary bypass times. Aortic root replacement, as opposed to repair, is indicated in patients with CTD, aortic root aneurysm > 4.5 cm, and extensive tissue loss^[35,36]^.

The management of type A dissections involving the aortic arch and descending aorta is variable, as such pathology can lead to a false lumen dilatation progressing to rupture, true lumen compression, and distal malperfusion despite successful management of the aortic root. The surgical options include hemiarch replacement, total arch replacement, and elephant trunk or frozen elephant trunk procedures^[37-40]^. A novel procedure is the Lupiae technique, which uses a graft with a bovine trunk at the proximal end dividing into three branches and a fourth branch for antegrade perfusion once the distal anastomosis is completed. The ascending aorta and aortic arch are replaced, and the supra-aortic vessels are reimplanted. This creates a Dacron landing zone for TEVAR procedure to be performed as a second step^[41]^. Alternatively, hybrid procedures include revascularisation of supra-aortic vessels followed by TEVAR. The supra-aortic vessels are bypass grafted and debranched creating a landing zone for the graft, this is followed by endovascular stent-grafting of the aortic arch and possibly the descending aorta^[42]^.

More recently, customised, curved, branched, and fenestrated stent grafts have been developed to make it easier to land the graft in the aortic arch obviating the need for supra-aortic revascularization. The pre-curved fenestrated grafts have a 95.8% operative success with a 30-day mortality of 1.6% for acute dissection and aortic arch aneurysms^[43]^.

#### Management of Type B Dissections

The initial management of a type B dissection is medical, with blood pressure control being crucial. In the subacute phase, between two weeks to three months, patients may benefit from endograft placement allowing for aortic remodeling^[44]^. The objective is to promote thrombosis of the false lumen by sealing the entry tear. The INvestigation of STEnt Grafts in Aortic Dissection (INSTEAD) trial showed improved five-year aorta specific survival and delayed disease progression in patients undergoing TEVAR in the subacute phase^[45]^. However, for chronic type B dissection, open surgery is an acceptable option, as in this group, TEVAR may not result in long-term benefit due to persistent pressurization of the false lumen because of distal fenestrations^[46]^.

#### TEVAR and its Practicality in Daily Practice

The current expert consensus recommends open surgery for thoracic aneurysm repair in patients with connective tissue diseases^[47]^. In such patients, the aortic wall is particularly fragile, and the radial force exerted by landing a stent graft can increase circumferential stress. The long-term radial force is a prerequisite for adequate anchor and sealing when deploying a stent graft.

The initial feasibility of TEVAR in CTD patients is supported by a few but limited studies. Together, 81% of the procedures were technically successful with the stent graft being landed proximally. The immediate complication rate is generally low, with a 1.9% risk of death, 1.9% risk of stroke, < 2% risk of spinal ischaemia, and 3.7% rate of conversion to open surgery. However, the longer-term outcomes are not fully reported yet and need further assessment with an appropriate follow-up period^[48]^.

The reintervention rate due to primary and secondary endoleaks is high and retrograde dissection can occur in patients undergoing TEVAR. The incidence of stent graft-induced re-entry tears is also significantly high, with 33% reported in Marfan patients compared to 3% in the general population and a mortality of nearly 30%^[49]^.

Ince et al.^[48]^ reported successful deployment of grafts in six MFS patients with chronic type B dissection and had zero 30-day mortality. Three patients required open conversion over a follow-up of 51 months and another developed retrograde false lumen flow.

Geisbüsch et al.^[49]^ used endovascular stent graft in eight CTD patients with a technical success of 88% (one primary endoleak). Three patients needed endovascular reintervention and half of the patients showed disease progression.

Nordon et al.^[50]^ reported a 100% technical success rate in seven MFS patients with chronic dissection. Two patients developed endoleaks and all patients showed continued dilatation of the thoracic aorta at 7 mm/year on follow-up imaging.

Marcheix et al.^[51]^ also achieved 100% technical success in 15 MFS patients. One patient had hemiplegic stroke. Primary endoleaks were observed in five patients.

Botta et al.^[52]^ used TEVAR in 12 patients who already had aortic root/arch surgery. Stent grafts were inserted urgently in five patients and electively in seven patients. There was no mortality or adverse neurological event, but one patient had open conversion due to persistent endoleak at three months and two patients showed dissection progression over 24 months.

Waterman et al.^[53]^ evaluated 15 MFS patients treated with TEVAR. Fifty percent of them had primary treatment failure in terms of type 1 endoleak, persisting flow in false lumen, or need for further interventions.

Eid-Lidt et al.^[54]^ evaluated 10 MFS patients with a 100% technical success rate; one patient died and another had transient ischaemic attack. The rate of secondary endoleak was 44% and reintervention rate was 33%. Cumulative mortality at 59-month follow-up was 20%. Long-term survival (at eight years) was 80%. Positive remodeling occurred in 37.5% of cases.

On the contrary to MFS, endovascular repair is avoided and not recommended in LDS patients and this is primarily due to the young age, rarity of condition, and extensive aortic disease. However, Kalra et al.^[55]^ reported that two patients with LDS and contained ruptures underwent successful endovascular repair, but follow-up is not available. 

A summary of some of the available evidence is shown in Table 1^[48-55]^ Endovascular repair is also not recommended for vascular EDS. In this cohort, even invasive angiography, unless absolute necessary, is to be avoided due to the high risk of vessel rupture, with one study showing a 67% complication rate and 12% mortality rate for digital subtraction angiography^[56]^.

**Table 1:** Summary of the evidence of TEVAR in patients with connective tissue disorders.

Study	Year	Number of patients	CTD type	Pathology	Morbidity/ reintervention	Mortality
Ince et al.^[48]^	2005	6	MFS	Dissection	33.3% - Elective open conversion	None
Geisbüsch et al.^[49]^	2008	8	MFS - 75%; EDS - 25%	Aneurysm - 12.5%	37.5% - Endoleak	None
				Dissection - 87.5%		
Nordon et al.^[50]^	2009	7	MFS	Aneurysm and dissection	33.3% - Endoleak	14% (30 days)
					83.3% - False lumen	
Marcheix et al.^[51]^	2008	15	MFS	Aneurysm and dissection	66.6% - Endoleak	20% (30 days)
Botta et al.^[52]^	2009	12	MFS	Dissection	8.3% - Endoleak	None
					16.6% - Disease progression	
Waterman et al.^[53]^	2012	16	MFS	Aneurysm and dissection	44% - Reintervention	25% (overall)
Eid-Lidt et al.^[54]^	2013	10	MFS	Dissection (type B)	40% - Endoleak	20% (overall)
					70% - Reintervention (overall)	
Kalra et al.^[55]^	2015	2	LDS	Aneurysm and dissection	None	None

CTD=connective tissue disorder; EDS=Ehlers-Danlos syndrome; LDS=Loeys-Dietz syndrome; MFS=Marfan syndrome; TEVAR=thoracic endovascular aortic repair

#### Open Repair and the Future Trend

There have been no large studies directly comparing open surgical repair and endovascular repair in CTD patients. However, open surgery for aneurysm repair is a well-established technique with a low complication rate as it has a 100% technical success rate. The perioperative mortality has been shown to range from 0 to 11.5%, with an overall survival rate of 53-96%^[57]^. The morbidity includes a risk of up to 6.5% for paraplegia, risk of up to 13% for permanent renal failure, and risk of up to 11% for re-exploration for bleeding^[57]^.

The complication rate in open surgery can be reduced by using extracorporeal partial left heart bypass, intraoperative cold renal perfusion, and cerebrospinal fluid drainage. It is also important to use branched grafts rather than visceral patches. In one review, visceral patches were used in 107 patients including 17 CTD patients^[58-60]^. Visceral patch aneurysm expansion occurred in eight patients, out of which three had CTD, which is a predisposing factor to patch aneurysm formation. The morbidity rate for open repair of patch aneurysm is 20-30%, which is high compared to the primary operation and endovascular repair^[61]^.

In comparison, endovascular surgery has a technical success rate ranging from 38% to 100% with a primary treatment failure rate of up to 44%. Treatment failure may occur due to type I or type II endoleak, retrograde dissection, and ongoing dilatation of the false lumen in chronic dissection. The aorta distal to the stent graft fails and patients may develop recurrent aneurysms and acute dissections. The mortality rate ranges from 0 to 25% with an overall survival of 75 to 100%, which is comparable to open surgery, although only few studies have been done on small cohorts^[55,61]^. This is encouraging as it shows endovascular therapy may safely be done in the short term, although concerns exist regarding longer-term outcomes and effectiveness. The perioperative morbidity includes a risk of paraplegia up to 3.3%, risk of permanent renal failure up to 6.7%, and risk of re-exploration for bleeding in up to 14% of patients^[62]^.

In view of the lower morbidity, endovascular therapy is possibly an option in patients who have an absurdly high risk of open surgery, such as elderly patients with multiple comorbidities^[63]^. However, patients with connective tissue diseases do not fall into this category as they tend to be young and otherwise healthy. As it is a simpler and quicker technique, TEVAR is also recommended in emergency situations, such as aortic rupture, where it can be life-saving^[64]^. Endovascular repair is especially useful when the proximal and distal landing zones exist and is recommended in such situations (*e.g*., focal intercostal patch aneurysm, as part of the frozen elephant trunk procedure or hybrid procedures where a synthetic graft has been inserted previously via open surgery)^[65^^-^68^]^. The complication rate from endovascular therapy can be reduced further by using ultrasound guidance to obtain vascular access.

TEVAR does have a limited role in the management and treatment of aortic disease in patients with CTD, however due to the tissue and vasculature of these patients causing technical difficulties, open surgical repair is still the preferred treatment option. TEVAR also does not have better outcomes than open surgical repair, which still means open repair is the preferred treatment option. TEVAR may be the preferred treatment in selective patient groups who are at high risk of mortality or increased morbidity from open surgery; with further advances and improving outcomes over time, TEVAR could offer an alternative to open surgery in patients with multiple comorbidities.

## CONCLUSION

Open repair remains the gold standard method of intervention in young patients with progressive CTD, especially in the setting of acute type A aortic dissection. However, TEVAR can be sought as a reliable alternative in emergency setting of diseases involving the descending thoracic aorta; yet the long-term data needs to be published to support such practice.

**Table:** 

Authors' roles & responsibilities	
AH SMAH BMO MUA	Substantial contributions to the conception or design of the work; or the acquisition, analysis, or interpretation of data for the work; drafting the work or revising it critically for important intellectual content; final approval of the version to be published Substantial contributions to the conception or design of the work; or the acquisition, analysis, or interpretation of data for the work; drafting the work or revising it critically for important intellectual content; final approval of the version to be published Substantial contributions to the conception or design of the work; or the acquisition, analysis, or interpretation of data for the work; drafting the work or revising it critically for important intellectual content; final approval of the version to be published Substantial contributions to the conception or design of the work; or the acquisition, analysis, or interpretation of data for the work; drafting the work or revising it critically for important intellectual content; final approval of the version to be published
